# Conjoined lumbosacral nerve root: a case report

**DOI:** 10.1186/s13256-024-04415-4

**Published:** 2024-03-07

**Authors:** Yasutaka Takagi, Hiroshi Yamada, Hidehumi Ebara, Hiroyuki Hayashi, Hiroyuki Inatani, Kazu Toyooka, Akari Mori, Yoshiyuki Kitano, Yasuji Ryu, Aki Nakanami, Tetsutaro Yahata, Hiroyuki Tsuchiya

**Affiliations:** 1 Department of Orthopedic Surgery, Tonami General Hospital, 1-61 Shintomi-Cho, Tonami City, Toyama, 939-1395 Japan; 2 Department of Radiology, Tonami General Hospital, 1-61 Shintomi-Cho, Tonami City, Toyama, 939-1395 Japan; 3 Department of Rehabilitation Medicine, Tonami General Hospital, 1-61 Shintomi-Cho, Tonami City, Toyama, 939-1395 Japan; 4 Department of Rehabilitation Medicine, Kanazawa University Hospital, 13-1 Takara-Machi, Kanazawa City, Ishikawa 920-8641 Japan; 5 Department of Orthopedic Surgery, Graduate School of Medicine, Kanazawa University, 13-1 Takara-Machi, Kanazawa City, Ishikawa 920-8641 Japan

**Keywords:** Conjoined nerve root, Partial laminectomy, Transforaminal lumbar interbody fusion

## Abstract

**Background:**

In patients with conjoined nerve roots, hemilaminectomy with sufficient exposure of the intervertebral foramen or lateral recess is required to prevent destabilization and ensure correct mobility of the lumbosacral spine. To the best of our knowledge, no case reports have detailed the long-term course of conjoined nerve roots after surgery.

**Case presentation:**

We report the case of a 51-year-old Japanese man with a conjoined nerve root. The main symptoms were acute low back pain, radiating pain, and right leg muscle weakness. Partial laminectomy was performed with adequate exposure to the conjoined nerve root. The symptoms completely resolved immediately after surgery. However, the same symptoms recurred 7 years postoperatively. The nerve root was compressed because of foraminal stenosis resulting from L5–S disc degeneration. L5–S transforaminal lumbar interbody fusion was performed on the contralateral side because of an immobile conjoined nerve root. At 44 months after the second surgery, the patient had no low back pain or radiating pain, and the muscle weakness in the right leg had improved.

**Conclusions:**

This is the first report of the long-term course of conjoined nerve root after partial laminectomy. When foraminal stenosis occurs after partial laminectomy, transforaminal lumbar interbody fusion from the contralateral side may be required because of an immobile conjoined nerve root.

## Background

Conjoined nerve root (CNR) is an embryological nerve root anomaly that primarily affects the lumbosacral region. Anomalous roots are primarily bifid, conjoined structures arising from a wide area of the dura. Due to their size and attachment to surrounding structures, these roots are uniquely susceptible to trauma. The effects of compression and entrapment are amplified in the presence of lateral recess stenosis, in which developmental changes and disc herniation reduce the available reserve space [1]. In patients with CNR, adequate exposure of the roots may be required to prevent persistent compression and reduce traction. Therefore, hemilaminectomy should be performed with sufficient exposure of the intervertebral foramen or lateral recess to prevent deleterious alterations in stability and ensure correct mobility of the lumbosacral spine [2]. To the best of our knowledge, no case reports have detailed the long-term course of CNR after surgery. Here, we report the long-term course of CNR in a patient who underwent partial laminectomy.

## Case presentation

A 51-year-old Japanese man with no relevant medical history visited a local doctor because of acute lower back pain and radiating pain in the right leg. He had no apparent history of trauma or other triggering events. He was employed, had no relevant family history, and had no history of tobacco smoking or alcohol consumption. He received a sacral epidural block but was referred to our department when it failed to provide relief from pain. Neurological examination revealed a positive straight-leg-raising test result at 80° on the right side. Manual muscle examination revealed weakness in the right leg (tibialis anterior, 3/5; extensor hallucis longus, 3/5; flexor hallucis longus, 4/5; and triceps surae, 4/5). The right patellar tendon reflex was normal, and the right Achilles tendon reflex was slightly diminished. Hypoesthesia was observed in the right L5 dermatome. Neurologically, these results indicate L5 nerve root impairment.

**Fig. 1:**
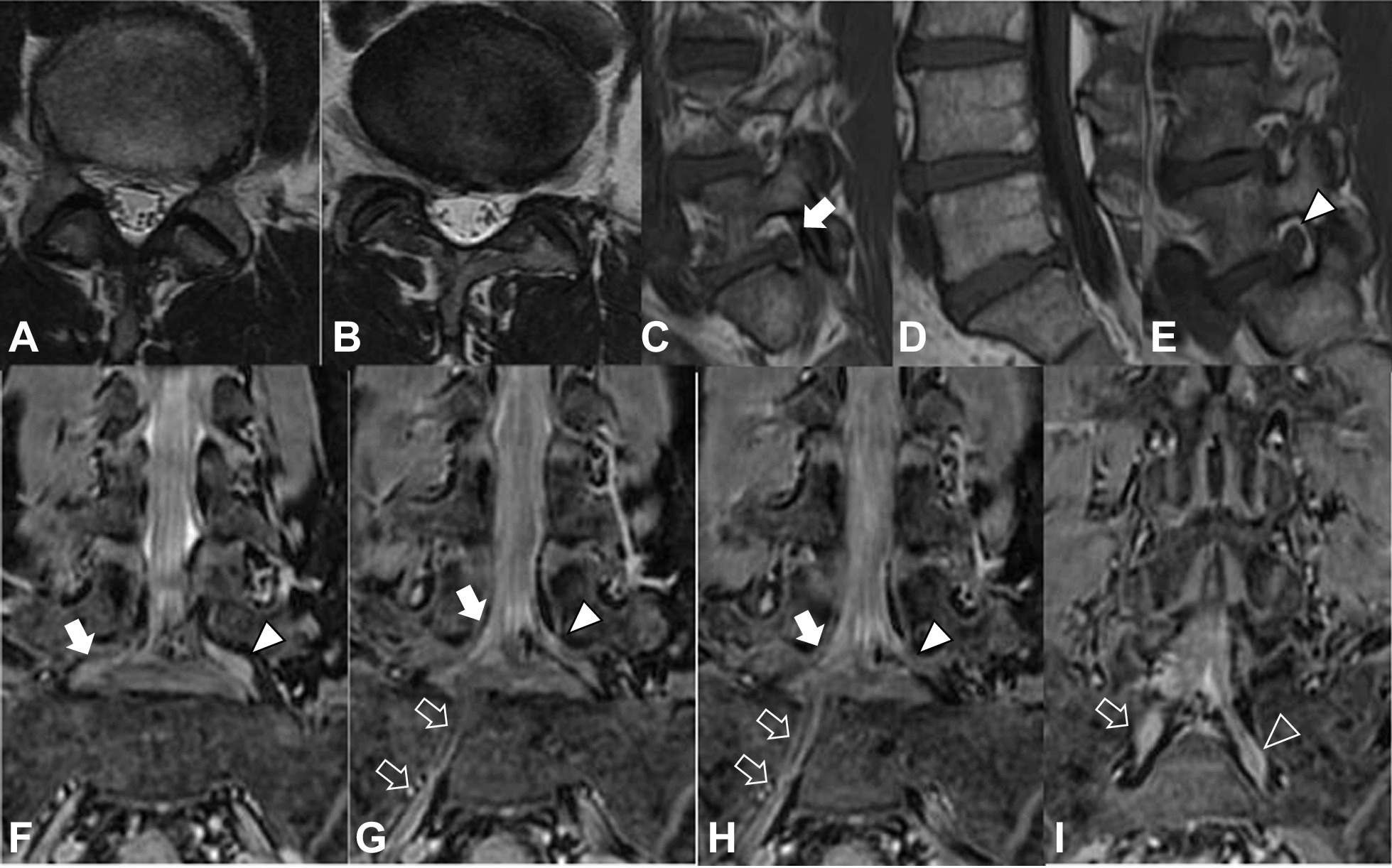
Magnetic resonance imaging findings showing no disc herniation on T2-weighted imaging (**a** L4–5 axial; **b** L5–S axial), no foraminal stenosis on T1-weighted imaging (**c** right sagittal; **d** central sagittal; **e** left sagittal), and a right conjoined lumbosacral nerve root (white solid arrow and white outline arrow) on T2-weighted imaging (**f**–**i** coronal). The right L5 nerve root (white solid arrow) is located significantly lower in the intervertebral foramen than the left L5 nerve root (white solid triangle) and joins a common trunk with the S1 nerve root (white outline arrow). The right S1 nerve root branches more proximally than the left S1 nerve root (white outline triangle). *MRI* magnetic resonance imaging

Magnetic resonance imaging (MRI) showed no disc herniation; however, a right conjoined lumbosacral nerve root was observed on T2-weighted imaging, and no foraminal stenosis was observed on T1-weighted imaging. The location of the right L5 nerve root in the intervertebral foramen was significantly lower than that of the left L5 nerve root, and the right L5 nerve root joined the common trunk with the S1 nerve root. The right S1 nerve root branched more proximally than the left S1 nerve root (Fig. 1).

**Fig. 2:**
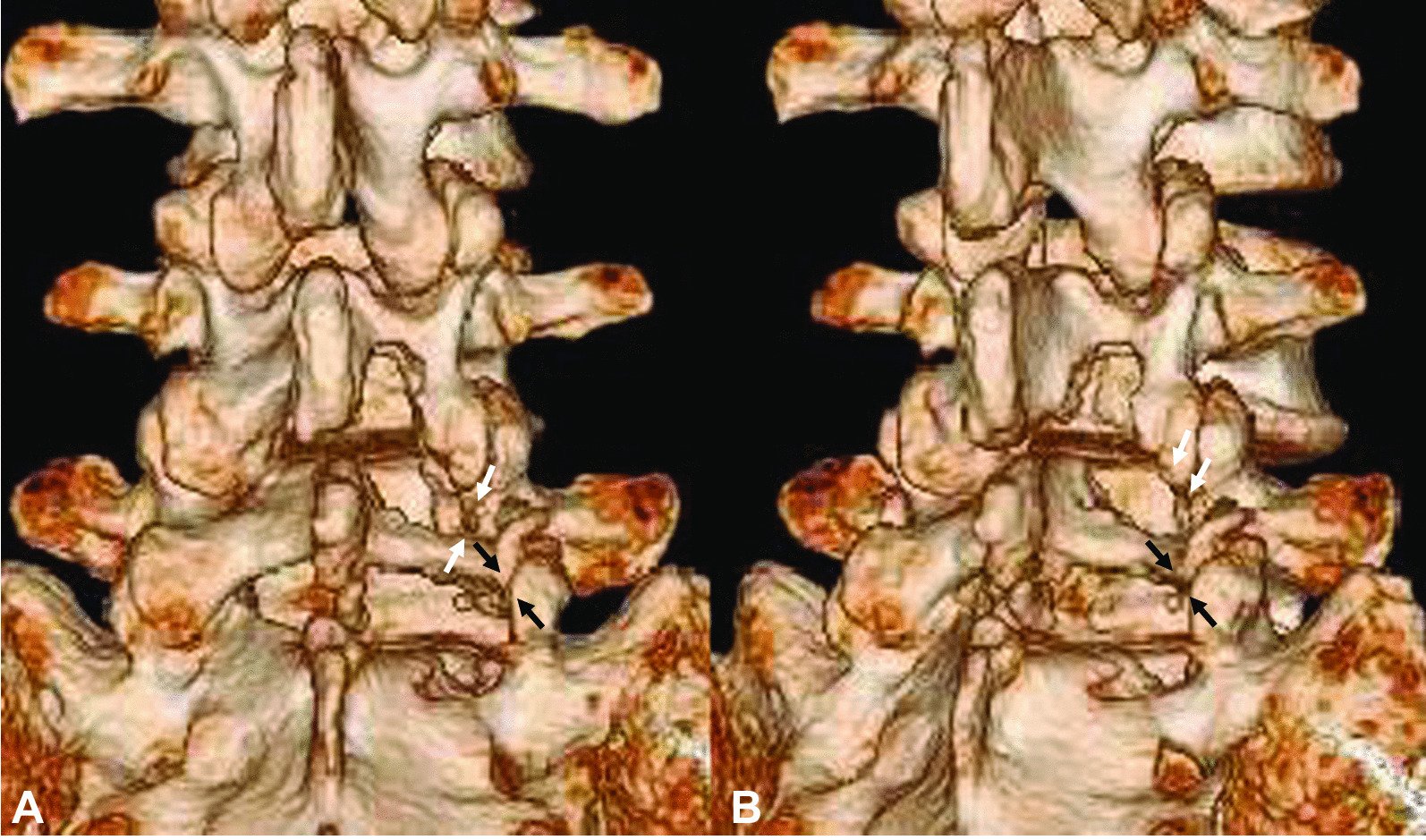
Postoperative 3D-CT demonstrating successful partial laminectomy (**a** posterior view; **b** oblique view). The right L4–5 facet joint (white arrow) and L5–S facet joint (black arrow) are partially resected. *3D-CT* three-dimensional computed tomography

**Fig. 3:**
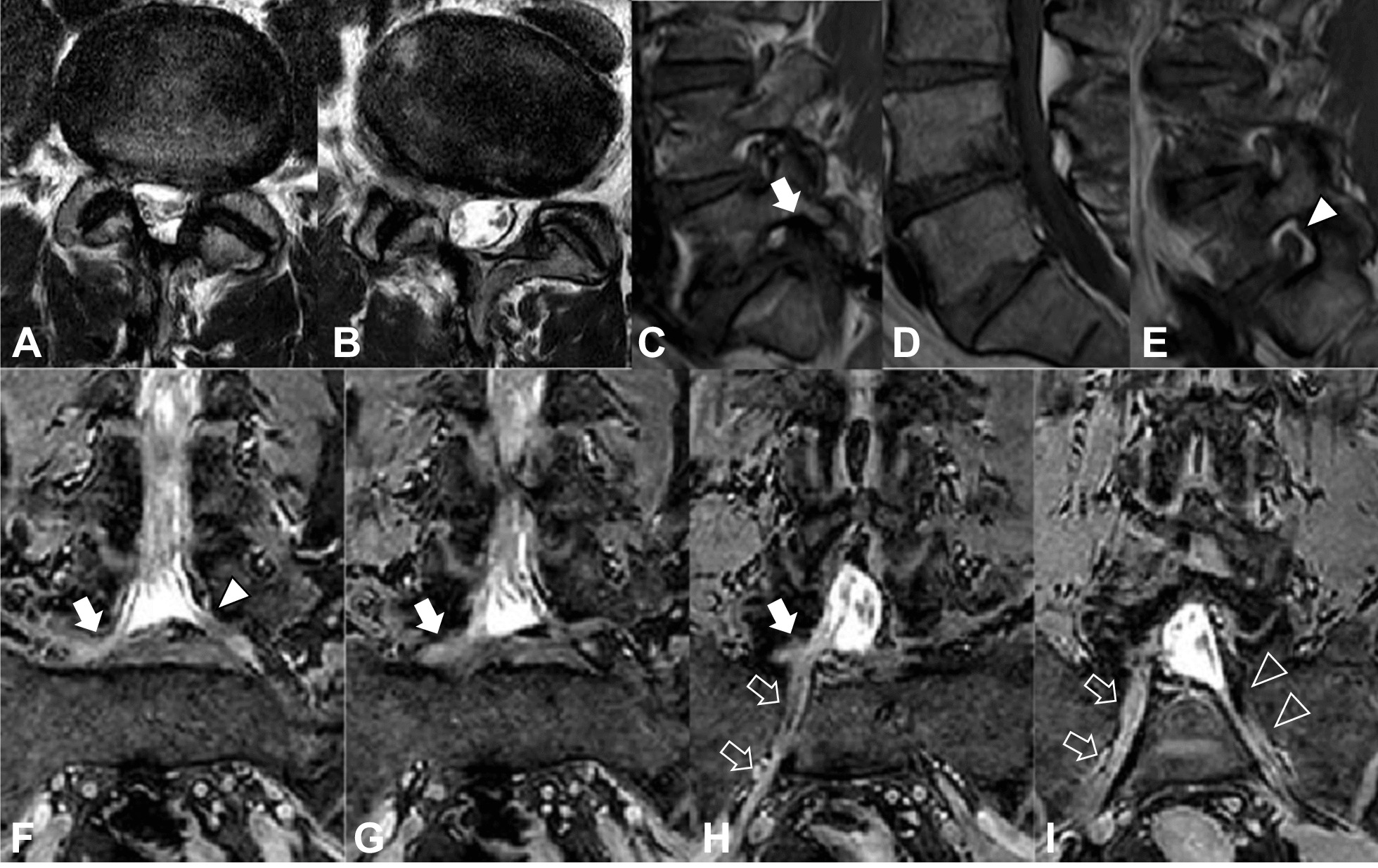
MRI showing no disc herniation on T2-weighted imaging (**a** L4–5 axial; **b** L5–S axial), right L5–S foraminal stenosis on T1-weighted imaging (**c** right sagittal; **d** central sagittal; **e** left sagittal), and right CNR (white solid arrow and white outline arrow) on T2-weighted imaging (**f**–**i** coronal). The right CNR (white solid arrow) is compressed due to right foraminal stenosis resulting from L5–S disc degeneration. *CNR* conjoined nerve root; *MRI* magnetic resonance imaging

**Fig. 4:**
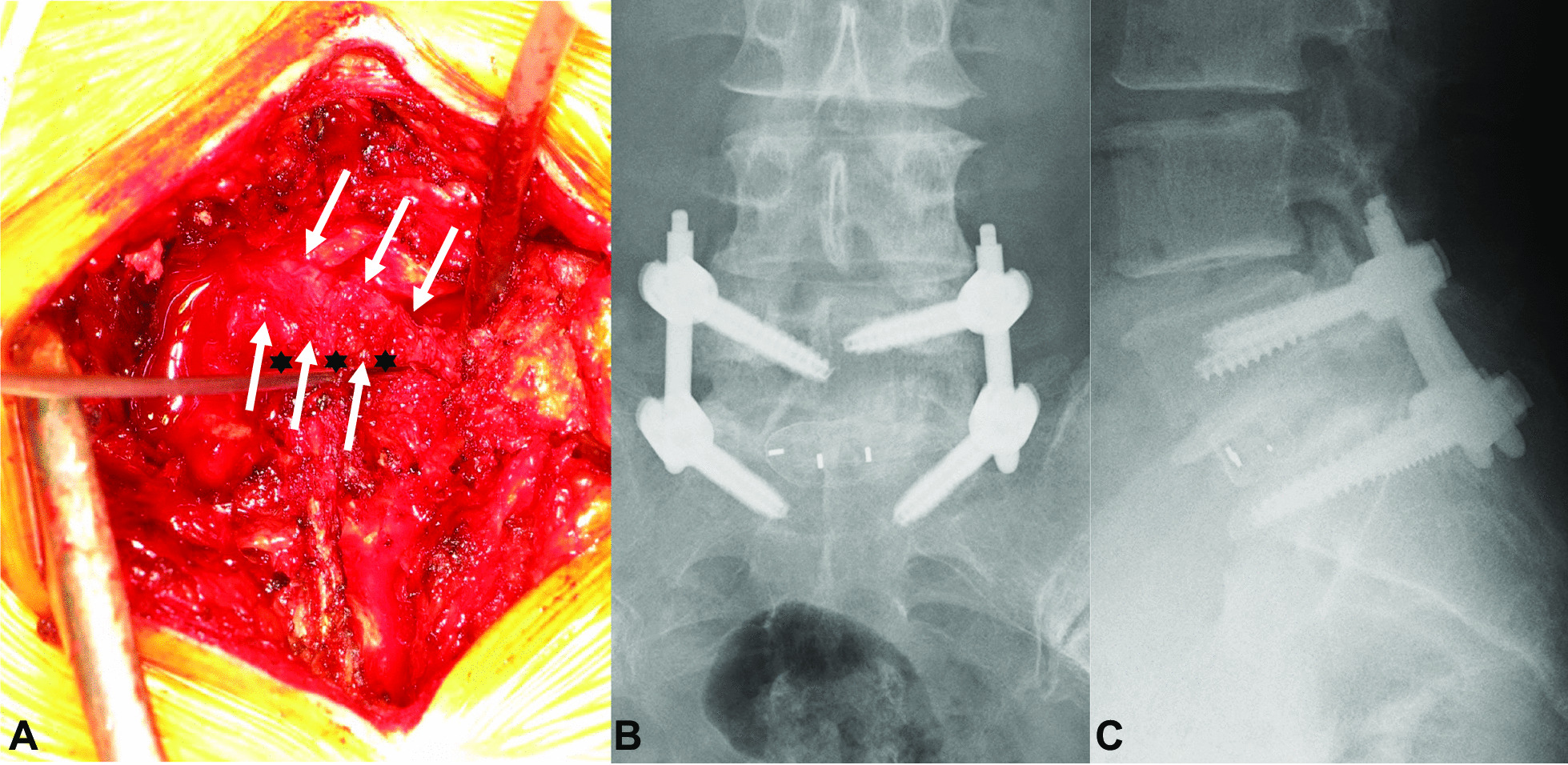
Right CNR (white arrow) crossing the L5–S disc space (heavy six-pointed black star). The right CNR is markedly swollen and cannot be safely mobilized (**a** intraoperative findings). Postoperative X-ray demonstrates successful L5–S TLIF (**b** AP view; **c** lateral view). *CNR* conjoined nerve root;* TLIF* transforaminal lumbar interbody fusion

He was diagnosed with CNR and administered analgesics and antiinflammatory drugs, and a selective nerve root block was performed. However, after 1 month, the pain did not improve, and we performed a partial laminectomy. Laboratory assessments showed no signs of an inflammatory reaction (C-reactive protein 0.09 mg/L, white blood cell count 4.0 × 10^9^/L, and platelet count 212 × 10^9^/L). Laboratory assessment of the liver and renal function showed no abnormalities [aspartate aminotransferase (AST) 29 U/L, alanine aminotransferase (ALT) 39 U/L, alkaline phosphatase (ALP) 170 U/L, blood urea nitrogen (BUN) 17.8 mg/dL, and creatinine 0.83 mg/dL]. Urine, serological, and microbiological analyses revealed no abnormalities. His body temperature was 36.7 °C, his pulse rate was 56 beats per minute, and his blood pressure was 125/69 mm/Hg at the time of admission. Intraoperatively, we discovered that the right L5 root originated from the caudal level of the L5 pedicle and was conjoined with the right S1 nerve root. The CNR was decompressed by resecting the facet joint. Postoperative three-dimensional computed tomography (3D-CT) demonstrated successful partial laminectomy. The right L4–5 and L5–S facet joints were partially resected (Fig. 2). Immediately after surgery, the symptoms completely resolved. However, the same symptoms recurred 7 years postoperatively. MRI did not show disc herniation on T2-weighted images, but T1-weighted images revealed right L5–S foraminal stenosis. The right L5 nerve root (the conjoined nerve root) was compressed owing to right foraminal stenosis from L5–S disc degeneration (Fig. 3). A neurological examination revealed a negative right straight-leg raising test. Manual muscle examination revealed weakness of the right leg (tibialis anterior, 3/5; extensor hallucis longus, 3/5). The right patellar and right Achilles tendon reflexes were normal. Hypoesthesia was observed in the right L5 dermatome. Neurologically, these results indicate an L5 nerve root impairment. He was diagnosed with right L5–S foraminal stenosis, administered analgesics and antiinflammatory drugs, and underwent a sacral epidural selective nerve root block. However, his pain did not improve after 3 months, and we performed L5–S transforaminal lumbar interbody fusion (TLIF). Laboratory assessments showed no signs of an inflammatory reaction (C-reactive protein 0.10 mg/L, white blood cell count 5.5 × 10^9^/L, and platelet count 204 × 10^9^/L). Laboratory assessments of liver and renal function showed no abnormalities (AST 28 U/L, ALT 38 U/L, ALP 176 U/L, BUN 18.4 mg/dL, and creatinine 0.81 mg/dL). Urine, serological, and microbiological analyses revealed no abnormalities. His body temperature was 36.5 °C, his pulse rate was 53 beats per minute, and his blood pressure was 106/72 mm/Hg at the time of the second admission. In the second surgery, a right L5–S facetectomy revealed that the right CNR crossed the L5–S disc space, and there was marked swelling of the right CNR that made it immobile. To perform TLIF, the right CNR must be safely retracted medially, the intervertebral disc should be removed, and a cage measuring 11 mm in height and 10 mm in width should be inserted into the L5–S disc space. We judged that if we created that space through surgical techniques, there would be a high risk of excessive traction and damage to the right CNR; therefore, we abandoned the insertion from the left side and performed TLIF from the contralateral side (Fig. 4). Immediately after surgery, the symptoms completely resolved.

At the time of writing this report, 44 months after the second surgery, the patient has no lower back or radiating pain, and the muscle weakness in the right leg has improved.

## Discussion and conclusions

In patients with conjoined nerve roots (CNRs), hemilaminectomy should be performed with sufficient exposure of the intervertebral foramen or lateral recess to prevent destabilization and ensure the correct mobility of the lumbosacral spine [2]. To the best of our knowledge, no case reports have detailed the long-term course of CNR after surgery.

The incidence of CNR is reportedly 2–17.3% [2–4]. The CNR is composed of two adjacent nerve roots sharing a common dural envelope after exiting the thecal sac [5]. Nerve root anomalies are known differential diagnoses of herniated intervertebral discs [2]. In recent case reports, CNR was not diagnosed using preoperative imaging during the management of CNR accompanied by lumbar disc herniation [6, 7]. Symptomatic undiagnosed CNR is a cause of failed back surgery because a simple discectomy without adequate decompression of the lateral recess and intervertebral foramen does not address the pathology [2, 5]. Furthermore, an inadvertent nerve root injury or battered nerve root syndrome can occur if a second nerve is not observed in the field. The pathoanatomy of CNR resulted from the focal stenosis of the lateral recess and intervertebral foramen. Creating room for increased nerve tissue in the root exit zone and intervertebral foramen is of central importance during treatment. Several surgical procedures, such as pediculectomy and facetectomy, have been described as treatments for CNR [1, 5, 8–14]. These treatment approaches aim to achieve adequate decompression. Lumbar root anomalies have been defined in detail by Neidre and MacNab [10]. Three groups were defined on the basis of the nerve position at the exit of the dura. Type 1A involves CNR originating from the rostral nerve root rather than the thecal sac. Type 1B involves CNR arising from the thecal sac near the caudal nerve root. Type 2A involves two separate nerve roots exiting the same neural foramen, leaving one neural foramen unoccupied. Type 2B involves two nerve roots exiting one neural foramen, with all the other foramina occupied. Finally, Type 3 involves two adjacent nerve roots with an anastomosis between them. According to this classification system, our case was Type 1B, with the cranial nerve root exiting at a right angle to the dural sheath, similar to the cervical nerve root that exits the dural sheath. In the second surgery, the right CNR crossed the L5–S disc space, and there was marked swelling of the right CNR that made it immobile. To perform TLIF, the right CNR must be safely retracted. We judged that if we created that space through surgical techniques, there would be a high risk of excessive traction and damage to the right CNR; therefore, we abandoned the insertion from the left side and performed TLIF from the contralateral side.

With advances in diagnostic modalities, reports on CNR are increasing. MRI-based diagnosis of CNR is based on indirect imaging signs, such as dural asymmetry (corner sign), atypical extradural fat (fat crescent sign), or angulation of the exiting nerve root (parallel sign) [15]. These indirect signs are non-specific and can be mimicked by disc herniation or extradural lesions [16]. However, newer diagnostic tools, such as MR neurography [17] and oblique lumbar MRI [18], can directly demonstrate CNR.

To the best of our knowledge, there are no case reports that detail the long-term course after surgery for CNR. Our patient underwent partial laminectomy with adequate exposure of the CNR. The symptoms resolved effectively and promptly after surgery. However, the same symptoms recurred 7 years postoperatively. The CNR was compressed due to foraminal stenosis resulting from L5–S disc degeneration. L5–S TLIF was performed on the contralateral side because of the immobile CNR. The patient had no low back or radiating pain, and the muscle weakness in the right leg improved for at least 44 months after the second surgery.

In our second surgery, TLIF was performed from the contralateral side because of the immobile CNR. Herein, we report the first case describing the long-term course of CNR after partial laminectomy with adequate exposure. When foraminal stenosis occurs after partial laminectomy, TLIF from the contralateral side may be required in cases of an immobile CNR.

Further studies with larger sample sizes are required to elucidate this crucial topic.

## Data Availability

Medical imaging data will not be shared because it is not fully anonymous.

## Abbreviations

3D-CT: Three-dimensional computed tomography
CNR: conjoined nerve root
MRI: magnetic resonance imaging
TLIF: transforaminal lumbar interbody fusion
